# Light-regulated templated self-assembly of bilayered nanotoroids

**DOI:** 10.1039/d6sc02354a

**Published:** 2026-04-22

**Authors:** Kintaro Miyamoto, Sota Mihara, Hiroki Itabashi, Sougata Datta, Hiroki Hanayama, Shiki Yagai

**Affiliations:** a Division of Advanced Science and Engineering, Graduate School of Engineering, Chiba University 1-33 Yayoi-cho, Inage-ku Chiba 263-8522 Japan; b Institute for Advanced Academic Research (IAAR), Chiba University 1-33 Yayoi-cho, Inage-ku Chiba 263-8522 Japan yagai@faculty.chiba-u.jp; c Department of Applied Chemistry and Biotechnology, Graduate School of Engineering, Chiba University 1-33 Yayoi-cho, Inage-ku Chiba 263-8522 Japan

## Abstract

Controlled molecular self-assembly driven by noncovalent interactions provides access to ordered architectures beyond the molecular scale. Yet the precise construction of higher-order assemblies remains difficult because the directionality of noncovalent interactions progressively decreases as structural hierarchy increases. Here we show that bilayered toroids can be generated by introducing *trans*-azobenzene units into a scissor-shaped diphenylnaphthalene dyad that otherwise forms toroidal assemblies. Rapid evaporation of a toluene solution of the azobenzene-appended dyad affords monolayered toroids, whereas slow evaporation yields bilayered toroids, indicating that the *trans*-azobenzene unit promotes the formation of a second toroidal layer. Consistent with their distinct dimensions, both monolayered and bilayered toroids organize on a substrate into two-dimensional hexagonal arrays with distinct lattice parameters, while the bilayered toroids further exhibit stacking along the *z* axis. In a less polar medium, this hierarchical stacking proceeds to give corrugated cylinders composed of approximately 10 bilayered toroids. The role of azobenzene units in forming the bilayered structure can be demonstrated by slow evaporation of a toluene solution of the photogenerated *cis* isomer, which affords only monolayered toroids, supporting the conclusion that the *trans*-azobenzene unit serves as a structural scaffold for bilayer formation.

## Abstract 2

A toroid-forming dyad bearing *trans*-azobenzene units forms bilayered nanotoroids *via* template-guided second ring closure, while photoisomerization to the *cis* form suppresses bilayer formation.

## Graphical abstract

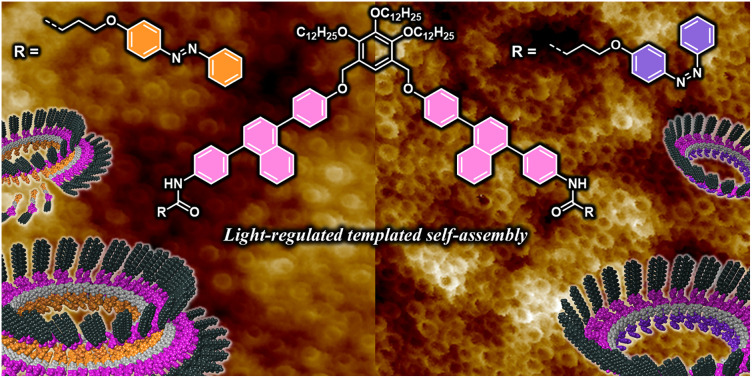

## Introduction

Molecular self-assembly gives rapid access to large and discrete structures that are difficult to reach by covalent synthesis because many weak interactions can act cooperatively during growth.^1–4^ In contrast to covalent synthesis, however, it remains challenging to control connectivity in self-assembly, that is, to define which contacts form and in what order, as an assembly develops.^5,6^ This becomes more difficult in hierarchical systems, where different noncovalent interactions are averaged and the interactive sites that direct further growth are hard to identify, and it becomes unclear where specific binding occurs and how growth directionality is defined.^7–9^ The problem is especially clear for closed objects such as self-assembled nanorings (toroids).^10–13^ Once a toroid has formed, it is still difficult to use it as a template for further growth because the highly directional end interactions that drive primary assembly are no longer available.^14–16^ Although toroid-edge-templated secondary nucleation has produced concentric toroids in a few systems,^17,18^ general molecular design rules remain limited. A practical solution would be to separate, within a single molecule, the interactions that form the primary toroid from those that drive secondary growth.^19^

**Fig. 1:**
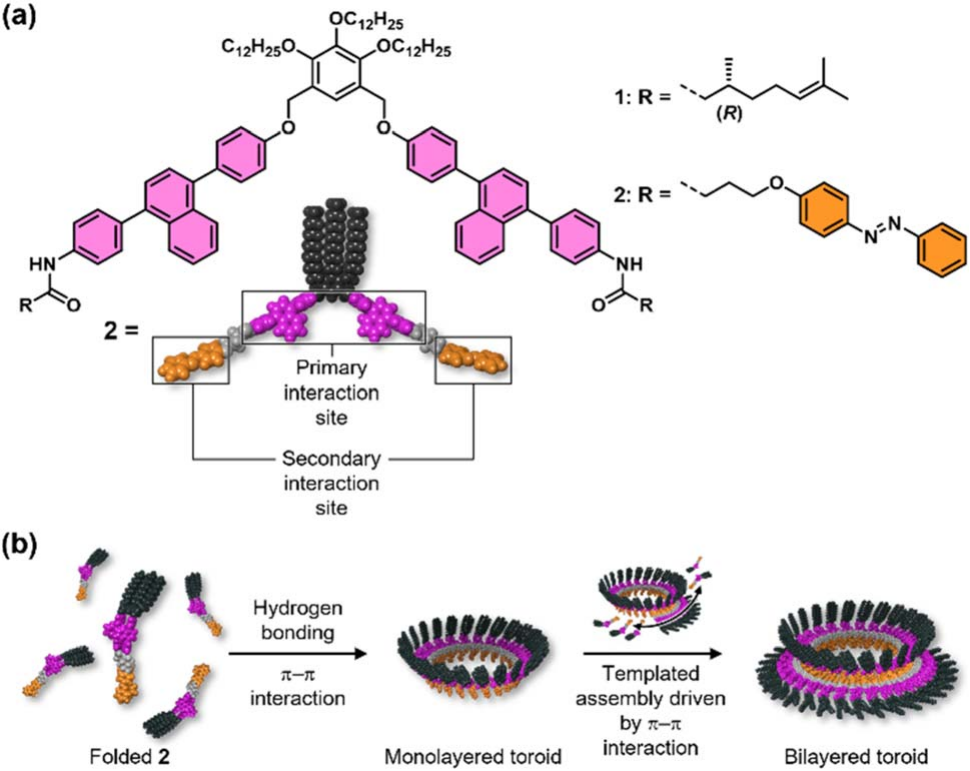
(a) Molecular structures of scissor-shaped diphenylnaphthalene dyads 1 and 2. (b) Schematic representation of the self-assembly of 2.

Our previous work established curvature-programmed dyads as a route to primary toroids.^20–23^ In these systems, intramolecular folding of scissor-shaped dyads preorganizes a bent conformation, and subsequent supramolecular polymerization proceeds with a defined curvature. A scissor-shaped azobenzene dyad, for example, forms discrete toroids and, under suitable conditions, further assembles into nanotubes through interactions between azobenzene units exposed on the toroid surface. However, this system also illustrates a limitation of such designs: introducing additional interaction sites into the side chains can perturb the delicate balance required for toroid formation, leading instead to elongated fibers.^24,25^ By contrast, replacing the azobenzene segment with diphenylnaphthalene in 1 introduces competition between ring closure and open-chain growth, giving both toroids and helical coils (Fig. 1a).^26^ This behavior suggests that the diphenylnaphthalene segment has a stronger tendency to sustain supramolecular growth than the azobenzene segment while still allowing curved assembly.

These observations led us to design 2, in which the functions required for primary toroid formation and secondary growth are spatially separated within a single molecule (Fig. 1a). The diphenylnaphthalene-based scaffold retains the interactions needed for curved growth and primary ring closure, whereas the terminal *trans*-azobenzene unit provides an additional peripheral interaction site for secondary growth around a preformed inner toroid. Thus, unlike previous toroid-based systems in which the same structural motif largely governs both toroid formation and further assembly, molecule 2 was designed to decouple primary toroid formation from subsequent higher-order growth. Depending on the drying conditions of its toluene solutions, 2 affords either monolayered or bilayered toroids (Fig. 1b). In a less polar solvent, bilayered toroids form directly in solution and then stack hierarchically to yield corrugated nanotubes as a precipitate. The idea that the azobenzene units exposed on the primary toroid act as anchoring sites for the formation of the second layer is supported by the finding that the *cis* isomer, generated by photoisomerization, gives only monolayered toroids. The present study thus shows that, if a molecule is designed to produce a single, well-defined mesoscale structure in the first instance, that structure can in turn serve as the basis for constructing more complex architectures at the next hierarchical level.

## Results and discussion

Compound 2 (for synthesis and characterization, see the SI) exists in a monomeric state in toluene. Upon cooling a toluene solution of 2 (*c* = 500 µM) from 100 to 20 °C at a rate of 1 °C min^−1^, UV-vis absorption spectra showed only a marginal change in the overlapping π–π* transitions of the naphthalene and *trans*-azobenzene moieties from *λ*_max_ = 327 to 329 nm (Fig. S1). Quantitative ^1^H NMR measurements in toluene-*d*_8_ indicated that 2 (*c* = 500 µM) is completely monomeric at 20 °C (Fig. S2). Additionally, dynamic light scattering (DLS) measurements showed no significant scattering arising from nanoscopic aggregates, further confirming the monomeric state of 2 in toluene at 20 °C (Fig. S3).

**Fig. 2:**
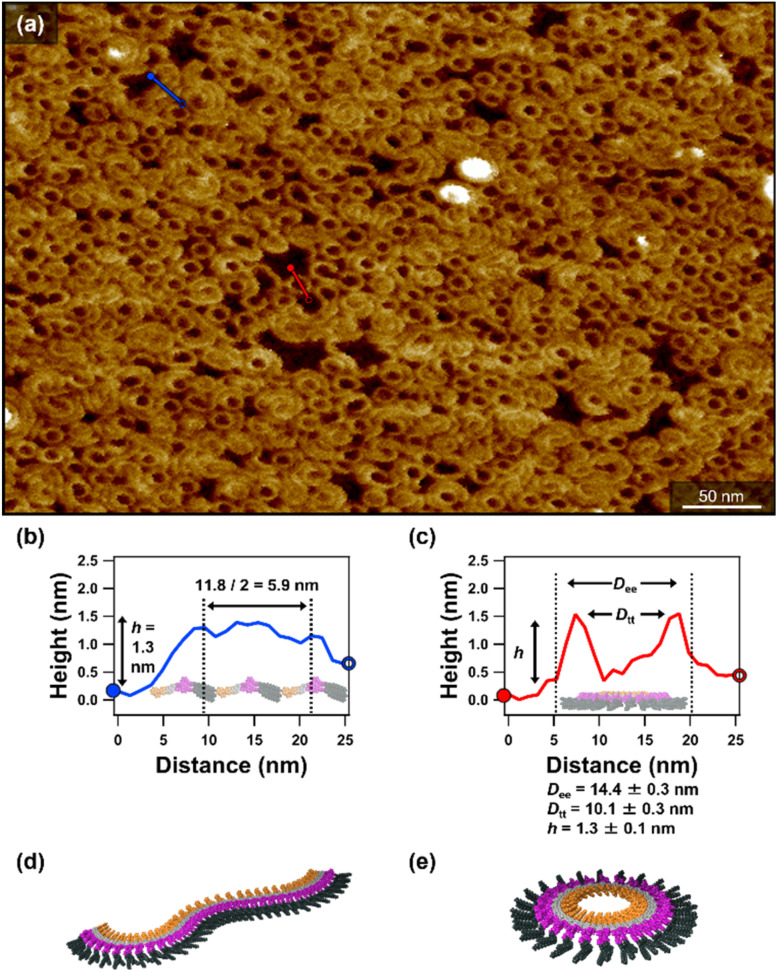
(a) AFM image of curved fibers and monolayered toroids formed by spin-coating a toluene solution of 2 (*c* = 500 µM) after cooling from 100 to 20 °C at a rate of 1 °C min^−1^. (b and c) AFM cross-sectional analyses of (b) the curved fibers (along the blue line in (a)) and (c) the monolayered toroids (along the red line in (a)). Average edge-to-edge diameters (*D*_ee_), top-to-top diameters (*D*_tt_) and heights (*h*) were determined from 100 monolayered toroids. The errors (±) represent 95% confidence limits. (d and e) Schematic representations of (d) a curved fiber and (e) a monolayered toroid of 2.

Interestingly, when an aliquot of the toluene solution was spin-coated onto a highly oriented pyrolytic graphite (HOPG) substrate, atomic force microscopy (AFM) revealed cyclic and randomly meandering supramolecular polymers with a uniform curvature (Fig. 2a).^10–13^ This finding demonstrates that 2 undergoes curved supramolecular polymerization even under the kinetically controlled conditions imposed by rapid solvent evaporation.^3^ Based on AFM cross-sectional analyses, the curved fibers have a height (*h*) of 1.3 ± 0.1 nm and a width of 5.9 nm. Top-to-top diameter (*D*_tt_) and edge-to-edge diameter (*D*_ee_) of monolayered toroids were estimated as *D*_tt_ = 10.1 ± 0.3 nm and *D*_ee_ = 14.4 ± 0.3 nm, respectively (Fig. 2b–e and S4). These dimensions are comparable to those of the toroidal structures formed by 1 reported previously.^26^ This observation suggests that 2 self-assembles into toroidal structures with the azobenzene units pointing toward the inner pore.

In contrast, slow evaporation of an aliquot of the toluene solution of 2 on a substrate yielded bilayered toroids. Upon drop-casting the solution (*c* = 500 µM, 10 µL) onto an HOPG substrate, AFM imaging revealed bilayered toroids consisting of an outer toroid enclosing an inner one (Fig. S5). This observation indicates that the slower drying process, compared with spin-coating, allows hierarchical organization of 2. In contrast, compound 1, which lacks azobenzene units, did not form bilayered toroids under the same conditions (Fig. S6), highlighting the crucial role of azobenzene–azobenzene π–π interactions in stabilizing the bilayered structure (Fig. S7).

**Fig. 3:**
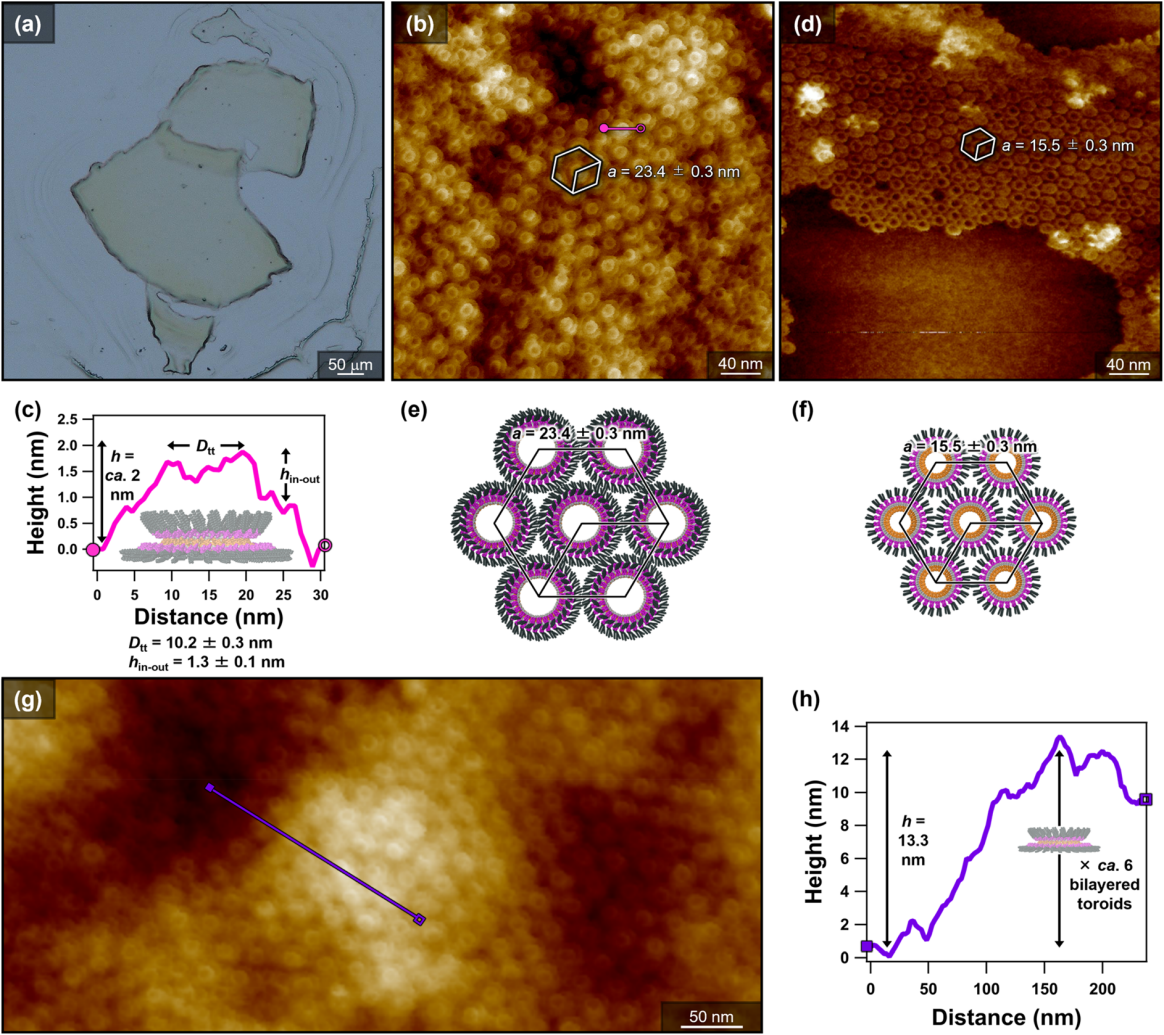
(a) Optical micrograph of the residual micro-sized sheets obtained by redispersion in toluene after drying a toluene solution of 2 (*c* = 500 µM) under a nitrogen flow. (b, d and g) AFM images of 2 upon redispersion in toluene, showing (b and g) bilayered toroids in the residual sheet region and (d) monolayered toroids in the dissolved region. Both monolayered and bilayered toroids exhibit hexagonal packing. The lattice parameters (*a*) for the bilayered and monolayered toroids were 23.4 ± 0.3 and 15.5 ± 0.3 nm, respectively. The average lattice parameters were determined from 100 each of the monolayered- and bilayered-toroid packings. (c and h) AFM cross-sectional analyses of (c) the bilayered toroids (along the pink line in (b)) and (h) the height difference of the surface of the residual sheet (along the purple line in (g)). Average top-to-top diameters (*D*_tt_) and height differences between the inner and outer toroids (*h*_in–out_) were determined from 100 bilayered toroids. (e and f) Schematic representations of the hexagonally packed (e) bilayered and (f) monolayered toroids (top views).

Based on the assumption that slower evaporation would promote the formation of the bilayered toroids, a toluene solution of 2 (*c* = 500 µM, 500 µL) was allowed to evaporate under a nitrogen flow for approximately 20 minutes, yielding a film. Upon addition of toluene (nominal *c* = 500 µM) and stirring, the film dispersed into thin sheets measuring several hundred micrometers across (Fig. 3a). AFM imaging of the sheet surface revealed the ordered packing of bilayered toroids (Fig. 3b). AFM cross-sectional analysis provided the structural parameters shown in Fig. 3c and S8. For the inner toroids, the top-to-top diameter (*D*_tt_) was estimated to be *D*_tt_ = 10.2 ± 0.3 nm, comparable to that of the monolayered toroids (see Fig. 2c). The height difference between inner and outer toroid (*h*_in–out_) was estimated as *h*_in–out_ = 1.3 ± 0.1 nm, which closely matches the thickness of the monolayered toroids (see Fig. 2c).

Notably, reflecting their size uniformity, both monolayered and bilayered toroids form two-dimensional hexagonal arrays on the substrate. The hexagonal lattice parameters (*a*) for the bilayered and monolayered toroids were 23.4 ± 0.3 and 15.5 ± 0.3 nm, respectively (Fig. S9). Thus, the bilayered toroids are only *ca.* 8 nm larger in lateral dimension than the monolayered toroids (Δ*a* = 7.9 nm; Fig. 3e and f). This increase is substantially smaller than twice the width of the curved fibers (2 × 5.9 nm), a value that would be expected for simple peripheral wrapping of an additional curved fiber around a pre-existing inner toroid. Instead, the observation supports a more three-dimensional bilayered architecture, in which the outer toroid encloses the inner toroid with partial lateral overlap.

AFM cross-sectional analysis over a several-hundred-nanometer scale showed a height difference of up to 13.3 nm (Fig. 3g and h), corresponding to approximately six bilayered toroids. Nevertheless, the hexagonal periodicity is maintained at the surface (Fig. S10), indicating regular stacking of the bilayered toroids along the *z* axis.

**Fig. 4:**
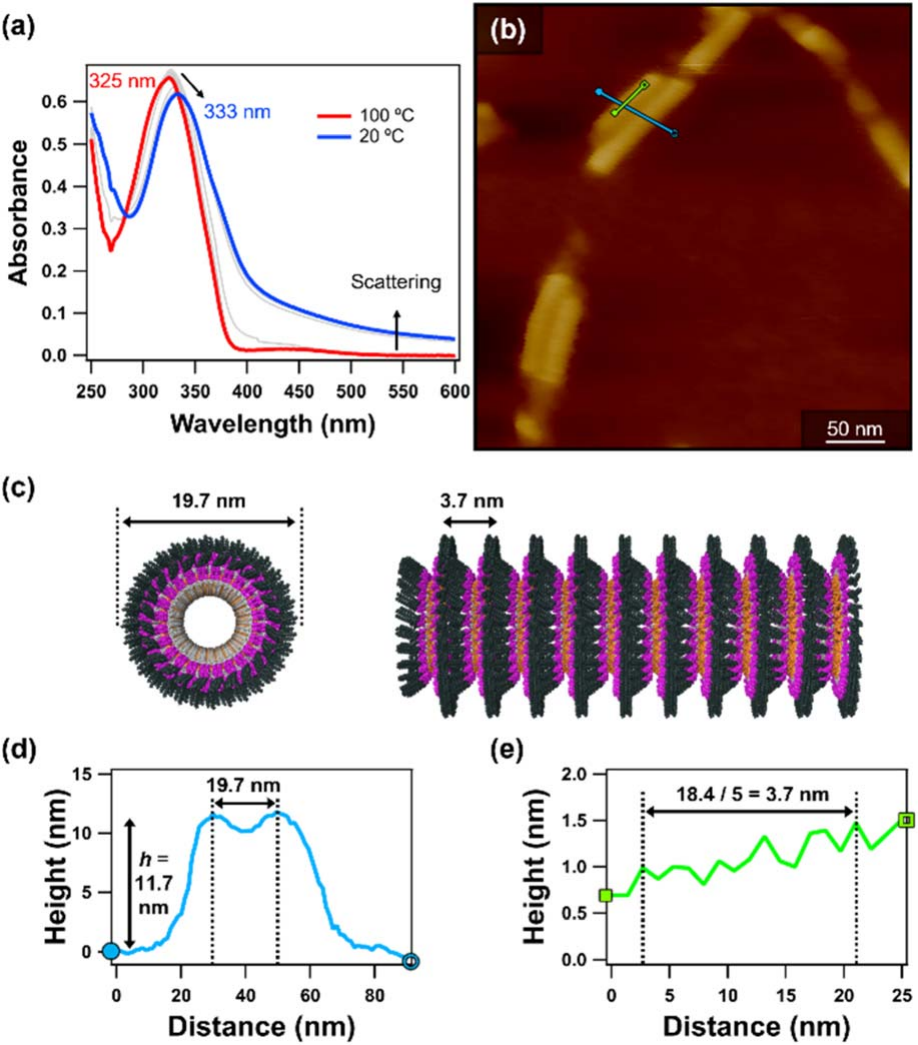
(a) UV-vis absorption spectra of 2 in MCH (*c* = 10 µM) upon cooling from 100 to 20 °C at a rate of 1 °C min^−1^. The temperature interval between spectra was 5 °C. (b) AFM image of cylinders of 2 with periodic surface corrugation prepared by spin-coating the MCH suspension. (c) Schematic representation of a cylinder of 2 with periodic surface corrugation. (d and e) AFM cross-sectional analyses along the (d) light-blue and (e) light-green lines in (b), respectively.

To gain further insight into the vertical stacking behavior of the bilayered toroids, the self-assembly of 2 was investigated in a non-polar solvent. A methylcyclohexane (MCH) solution of 2 (*c* = 10 µM, the highest concentration at which 2 is fully soluble at 100 °C) was cooled from 100 to 20 °C at a rate of 1 °C min^−1^. During this cooling process, the UV-vis absorption spectra exhibited a red shift of the overlapping π–π* transitions of the naphthalene and *trans*-azobenzene moieties from *λ*_max_ = 325 to 333 nm. This shift is consistent with interactions involving naphthalene and/or *trans*-azobenzene units (Fig. 4a). Around 30 °C, the baseline increased because of light scattering, accompanied by visible turbidity. AFM imaging of the assemblies obtained at 20 °C revealed *ca*. 100 nm-long cylinders with periodic surface corrugation (Fig. 4b and c).^27–32^ From the top-to-top distance between adjacent cylinders, the width was estimated to be 19.7 nm (Fig. 4d), comparable to the diameter of the bilayered toroids. AFM cross-sectional analysis along the long axis of the cylinders showed a periodic corrugation of 3.7 nm (Fig. 4e). During this process, an increase in absorbance at *λ* = 350 nm, originating from the absorption band of 2, was observed together with a simultaneous increase in apparent absorbance at *λ* = 590 nm, arising from baseline elevation due to light scattering (Fig. S11). Furthermore, the absence of isolated toroidal structures in the AFM images suggests that toroid formation and axial stacking occur in a coupled manner rather than as separable steps. These results support a hierarchical assembly pathway in which bilayered toroids stack regularly along the cylinder axis to generate periodically corrugated cylindrical superstructures.

**Fig. 5:**
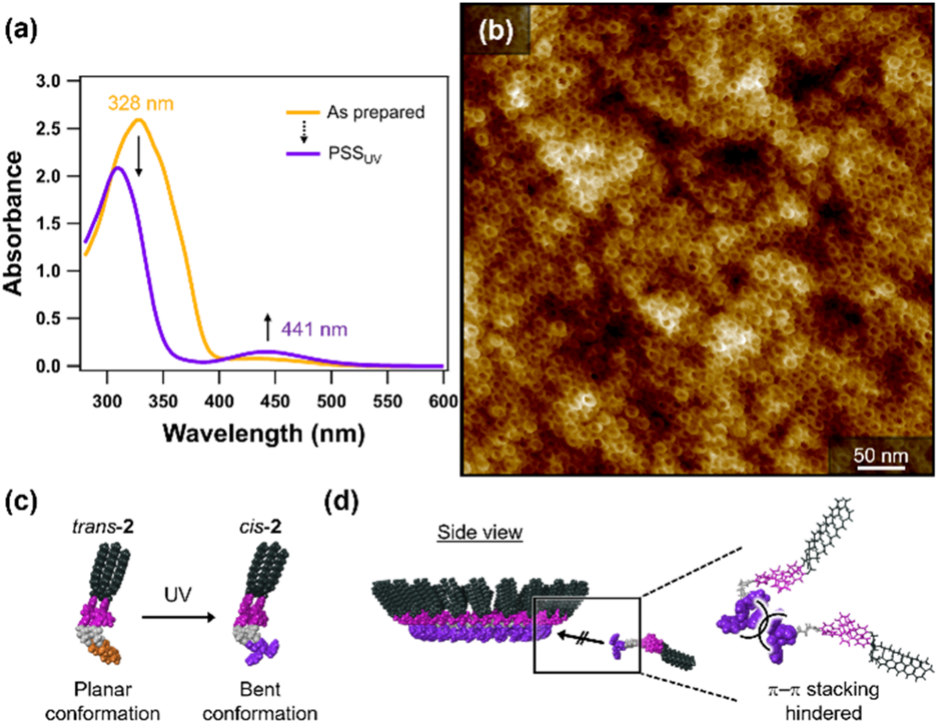
(a) UV-vis spectra of 2 in toluene (*c* = 500 µM) at 20 °C recorded at as-prepared (orange spectrum) and PSS_UV_ (purple spectrum). (b) AFM image of monolayered toroids of *cis*-2 (*c* = 500 µM) prepared by drop-casting the solution onto an HOPG substrate. (c and d) Schematic representations of (c) the *trans* → *cis* photoisomerization of 2 and (d) the suppression of the template-guided second closure event (*i.e.*, the bilayered-toroid formation) due to reduced π–π stacking of *cis*-2.

To probe the role of the *trans*-azobenzene units in bilayered-toroid formation, photoisomerization experiments were performed. When a monomeric solution of 2 in toluene (*c* = 500 µM) was irradiated with UV light (*λ* = 365 nm) for 5 min, a smooth *trans* → *cis* isomerization of the azobenzene moieties was confirmed by the attenuation of the band at *λ* = 328 nm and the growth of the band at *λ* = 441 nm (Fig. 5a). ^1^H NMR indicated the almost quantitative photoisomerization (Fig. S12). This toluene solution of *cis*-2 (*c* = 500 µM) was dried under a nitrogen flow to prepare a film. Upon re-addition of toluene, this film dissolved completely without leaving any residual sheet (*c* = 500 µM), suggesting that bilayered toroids had not formed. Furthermore, upon drop-casting the toluene solution of *cis*-2 onto HOPG, AFM revealed only monolayered toroids, with no evidence of bilayered toroids (Fig. 5b). Because the linkers spatially separate the azobenzene units from the diphenylnaphthalene and hydrogen-bonding units that drive primary toroid formation, 2 can form monolayered toroids even in its *cis*-isomeric form. Collectively, these results demonstrate that the *trans*-azobenzene moieties of 2 are essential for bilayered-toroid formation, acting as adhesive sites where aromatic–aromatic contacts between the planar *trans*-azobenzene units promote a template-guided cyclization of the outer toroid around the preformed inner toroid (Fig. 5c and d).

**Fig. 6:**
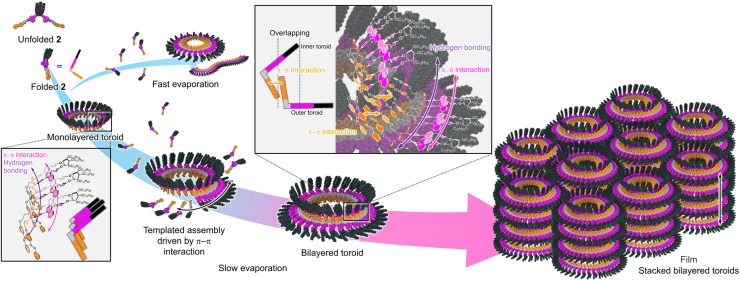
Schematic representation of the proposed mechanism for the formation of bilayered toroids by 2.

A proposed self-assembly mechanism of 2 is illustrated in Fig. 6. Molecule 2 adopts a folded conformation through intramolecular hydrogen bonding and π–π stacking of the diphenylnaphthalene units, and undergoes curvature-emergent self-assembly upon solvent evaporation to form monolayered toroids. When solvent evaporation is rapid, molecules that fail to complete ring closure instead form curved (open-ended) fibers. In contrast, under slower evaporation conditions, these species reorganize by using the planar *trans*-azobenzene units arranged on one face of the monolayered toroids as a scaffold for π–π interactions, leading to the formation of larger toroids and ultimately bilayered toroids. The formation of bilayered toroids was observed irrespective of the substrate (HOPG, silicon, mica, or glass) and casting solvent (toluene, chloroform, or THF) (Fig. S13), suggesting that this behavior is an intrinsic property of 2. Notably, further hierarchical organization—including vertical stacking into cylindrical structures and their lateral assembly into well-defined hexagonal packing—is observed exclusively for the bilayered toroids. This indicates that bilayered toroids possess a more robust and structurally uniform architecture than their monolayered counterparts.

## Conclusions

In conclusion, we have shown that appending an additional photoresponsive interaction site to a toroid-forming scaffold enables the programmed construction of a new higher-order toroid-based architecture, bilayered nanotoroids. Whereas the diphenylnaphthalene segment directs curved growth and primary ring closure, the *trans*-azobenzene unit promotes secondary organization around a preformed inner toroid, giving bilayered toroids that further organize into hexagonal arrays and stacked corrugated cylindrical superstructures. The suppression of bilayer formation upon *trans*-to-*cis* photoisomerization of the azobenzene units supports the view that the appended azobenzene unit acts as a structural scaffold for hierarchical growth. More broadly, these findings suggest a molecular design principle for hierarchical self-assembly: the interactions responsible for forming a primary self-assembled object can be separated from those that drive subsequent growth to the next structural level. In this sense, the toroid is transformed from a terminal product of self-assembly into a template for further organization. Because these bilayered toroids can be integrated into submillimeter-level sheet-like materials, this system may provide a platform for the development of larger porous materials with controlled transport, separation, and confinement properties. Future work will focus on preparing larger and more highly ordered films and on elucidating their structural and functional properties.

## Author contributions

S. Y., K. M. and S. M. designed the project. K. M. and S. M. performed all the experimental work. K. M. and S. Y. prepared the overall manuscript, including figures. All authors, including H. I., S. D. and H. H., contributed by revising and/or commenting on the manuscript. S. Y. supervised the overall research.

## Conflicts of interest

There are no conflicts to declare.

## Supplementary Material

SC-OLF-D6SC02354A-s001

## Data Availability

The data that support the findings of this work have been included in the main text and electronic supplementary information (SI). Supplementary information is available. See DOI: https://doi.org/10.1039/d6sc02354a.
