# Supplementary material for: Light-regulated templated self-assembly of bilayered nanotoroids
Source: Chem Sci. 2026 Apr 22;17(22):11201–6. doi: 10.1039/d6sc02354a (PMC13127164; doi:10.1039/d6sc02354a)
Supplement: SC-017-D6SC02354A-s001 [file SC-017-D6SC02354A-s001.pdf]

Supplementary Information (SI) for Chemical Science.
This journal is © The Royal Society of Chemistry 2026

Electronic Supplementary Information

Light-regulated templated self-assembly of bilayered nanotoroids

Kintaro Miyamoto,[a] Sota Mihara,[a] Hiroki Itabashi,[a] Sougata Datta,[b] Hiroki Hanayama[c] and Shiki Yagai*[b,c]

a. Division of Advanced Science and Engineering, Graduate School of Engineering, Chiba University, 1-33 Yayoi-cho, Inage-ku, Chiba 263-8522, Japan.
b. Institute for Advanced Academic Research (IAAR), Chiba University, 1-33 Yayoi-cho, Inage-ku, Chiba 263-8522, Japan.
c. Department of Applied Chemistry and Biotechnology, Graduate School of Engineering, Chiba University, 1-33 Yayoi-cho, Inage-ku, Chiba 263-8522, Japan.

E-mail: yagai@faculty.chiba-u.jp
Fax: +81-(0)43-290-3401
Tel: +81-(0)43-290-3169

Table of Contents

1. Materials and Methods..S2

2. Synthesis and Characterization..S3

3. Supporting Figures...S9

Fig. S1 UV–Vis absorption spectra of **2** in toluene S9
Fig. S2 ^{1}H NMR experiment of **2** in toluene-d_8........................ S9
Fig. S3 DLS measurements of **2** in toluene S10
Fig. S4 Size distributions of monolayered toroids of **2** S10
Fig. S5 AFM image of bilayered toroids of **2** S11
Fig. S6 UV–Vis absorption spectra and AFM images of monolayered toroids of **1** ... S12
Fig. S7 Comparison of self-assembly behaviors of **1** and **2**........................ S13
Fig. S8 Size distributions of bilayered toroids of **2** S14
Fig. S9 Lattice parameter distributions of mono- and bilayered toroids of **2**............ S14
Fig. S10 Large-area AFM image of bilayered toroids of **2**........................ S15
Fig. S11 UV–Vis absorption spectra of **2** in MCH S16
Fig. S12 Photoirradiation experiments and ^{1}H NMR study of **2** S17
Fig. S13 AFM images of bilayered toroids of **2** S18

4. Supporting References...S19

1. General.

Materials and Methods: All starting materials and reagents, purchased from commercial suppliers, were of reagent grade and used without further purification. Medium-pressure column chromatography was carried out with a Biotage Flash Purification System Isolera™ One using Biotage® Sfär Silica High-Capacity Duo 20 µm 25 g cartridges. The solvents used for the preparation of supramolecular assemblies were either spectroscopic grade or with a purity of at least 95.0%, which were used as received without further purification. Gel permeation chromatography (GPC) was performed with recycling preparative liquid chromatography (LC-9225NEXT, Japan Analytical Industry) equipped with two GPC columns (JAIGEL-2HR Plus and JAIGEL-2.5HR Plus). ^{1}H and ^{13}C nuclear magnetic resonance (NMR) spectra were recorded in $CDCl_3$ on Bruker AVANCE III-400M and Bruker AVANCE NEO 500 NMR spectrometers. Chemical shifts were reported in parts per million (ppm, δ) and were referenced to the chemical shifts of tetramethylsilane (TMS) at 0.00 ppm. The resonance multiplicity is described as s (singlet), d (doublet), t (triplet), and m (multiplet). High-resolution mass spectra (HRMS) were measured on an Orbitrap Exploris 120 (Thermo Fisher Scientific) using electrospray ionization (ESI). UV–Vis absorption spectra were recorded on a JASCO V760 spectrophotometer equipped with a JASCO ETCS-761 temperature-control unit. These spectra were recorded using a screw-capped quartz cuvette of 1.0 mm pathlength. Dynamic light scattering (DLS) measurements were conducted on Zetasizer Nano (Malvern Instruments) under 4.0 mW He-Ne laser (λ = 633 nm). The scattering angle was set at 173°. Molecular mechanics calculations were performed on MacroModel/Maestro version 12.2 (Schrödinger) with OPLS2005 force field without solvent.

Atomic force microscopy (AFM): AFM images were obtained under ambient conditions using a Multimode 8 Nanoscope V (Bruker AXS) in PeakForce Tapping (ScanAsyst) mode. The scan rate was set to 0.996 Hz. Silicon cantilevers (SCANASYST-AIR) with a spring constant of 0.4 N/m and a frequency of 70 kHz (nominal value, Bruker, Japan) were used. The samples were prepared by spin-coating (3000 rpm, 1 min) approximately 10 µL of solutions of assemblies onto freshly cleaved highly oriented pyrolytic graphite (HOPG, 0.5 cm × 0.5 cm), silicon, mica (1.0 cm × 1.0 cm) and glass at room temperature. AFM images were processed using NanoScope Analysis 3.0 (Bruker).

2. Synthesis and Characterization

Compound **2** was synthesized by the following procedure (Scheme S1). Compound **6** was purchased from commercial suppliers and used without further purification. Synthesis of **3** was reported previously.[S1]

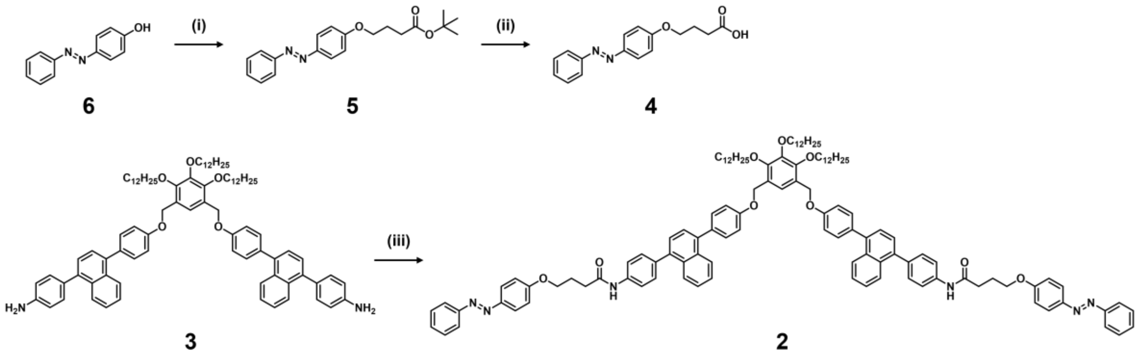

Scheme S1. Synthesis of **2**. Reagents and conditions: (i) tert-butyl 4-bromobutanoate, K_2CO_3, DMF, 65 °C; (ii) trifluoroacetic acid, CH_2Cl_2, 0 °C → r.t.; (iii) **4**, EDC·HCl, DMAP, CH_2Cl_2, r.t..

Synthesis and characterization of 5: Compound **6** (396 mg, 2.00 mmol) was added to a suspension of K_2CO_3 (832 mg, 6.02 mmol) in dry DMF (5 mL) at 65 °C. To this mixture, a solution of tert-butyl 4-bromobutanoate (497 mg, 2.23 mmol) in dry DMF (5 mL) was added dropwise, and the reaction mixture was stirred at 65 °C under N_2 atmosphere for 1 h. After cooling to room temperature, the reaction mixture was neutralized by adding aqueous HCl (2 M), and then diluted with a mixture of ethyl acetate and *n*-hexane (1:4). The resulting solution was washed with water and brine. The organic layer was separated, dried over Na_2SO_4, and then evaporated to dryness under reduced pressure. The crude product was purified by column chromatography over silica gel (eluent: ethyl acetate: *n*-hexane = 1:1, v/v) to give pure compound **5** as an orange solid (586 mg, 86% yield).

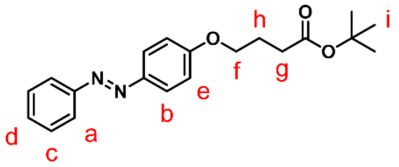

m.p. 59–61 °C. ^{1}H NMR (400 MHz, $CDCl_3$, 293K) δ 7.91 (d, J = 9.0 Hz, 2H, AzoH_a), 7.89–7.86 (m, 2H, AzoH_b), 7.52–7.48 (m, 2H, AzoH_c), 7.46–7.41 (m, 1H, AzoH_d), 7.00 (d, J = 9.0 Hz, 2H, AzoH_e), 4.09 (t, J = 6.2 Hz, 2H, -OCH_f), 2.46 (t, J = 7.3 Hz, 2H, -O_2CCH_g), 2.15–2.08 (m, 2H, CH_h), 1.46 (s, 9H, -CCH_i). ^{13}C NMR (125 MHz, $CDCl_3$, 323K) δ 172.33, 161.47, 152.93, 147.18, 130.22, 128.94, 124.69, 122.53, 114.80, 80.43, 67.33, 31.99, 28.13, 24.78. The peaks at δ 124.92, 122.55, 115.77, and 80.43 are assigned to *cis* **5**. HRMS (APCI): *m*/*z* calcd for $C_{20}H_{25}O_3N_2$ 341.1860 $[M+H]^+$, found 341.1856.

Synthesis and characterization of 4: Compound **5** (202 mg, 0.593 mmol) was dissolved in dry CH_2Cl_2 (3 mL) and the resulting solution was stirred at 0 °C for 5 min. To this, trifluoroacetic acid (3358 mg, 29.5 mmol) in dry CH_2Cl_2 (1 mL) was added dropwise at 0 °C, and then the resulting mixture was stirred at room temperature for 1 h. The reaction mixture was evaporated *in vacuo* to dryness to give compound **4** as a yellow solid (185 mg). This product was found to be almost pure and used for the next reaction without further purification.

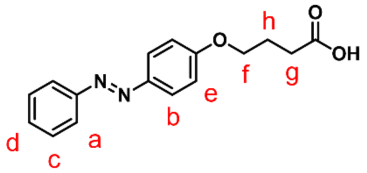

m.p. 59–61 °C. ^{1}H NMR (400 MHz, $CDCl_3$, 293K) δ 7.92 (d, J = 9.0 Hz, 2H, AzoH_a), 7.89–7.87 (m, 2H, AzoH_b), 7.53–7.48 (m, 2H, AzoH_c), 7.46–7.42 (m, 1H, AzoH_d), 7.00 (d, J = 9.1 Hz, 2H, AzoH_e), 4.13 (t, J = 6.0 Hz, 2H, -OCH_f), 2.64 (t, J = 7.2 Hz, 2H, $HO_2CC$$H_g$), 2.21–2.15 (m, 2H, C$H_h$). ^{13}C NMR (125 MHz, $CDCl_3$, 323K) δ 178.12, 161.26, 152.91, 147.27, 130.28, 128.96, 124.72, 122.56, 114.78, 69.97, 30.33, 24.40. HRMS (APCI): *m/z* calcd for $C_{16}H_{17}O_3N_2$ 285.1234 $[M+H]^+$, found 285.1229.

Synthesis and characterization of 2: Compound **3** (114 mg, 0.0892 mmol), **4** (114 mg, 0.401 mmol), 4-dimethylaminopyridine (DMAP, 3 mg, 0.0246 mmol) and 1-(3-dimethylaminopropyl)-3-ethylcarbodiimide hydrochloride (EDC·HCl, 105 mg, 0.548 mmol) were dissolved in dry CH_2Cl_2 (3 mL). The resulting mixture was stirred at room temperature under N_2 atmosphere for 22 h. The reaction mixture was neutralized by adding aqueous NaOH (1 M), and then diluted with ethyl acetate. The resulting solution was washed with water and brine. The organic layer was separated, dried over Na_2SO_4, and evaporated *in vacuo* to dryness. The crude product was purified by column chromatography over silica gel (eluent: ethyl acetate: *n*-hexane = 1:1, v/v) and further purified by GPC (eluent: $CHCl_3$) to give pure compound **2** as an orange solid (51 mg, 32 % yield). m.p. 150–154 °C.

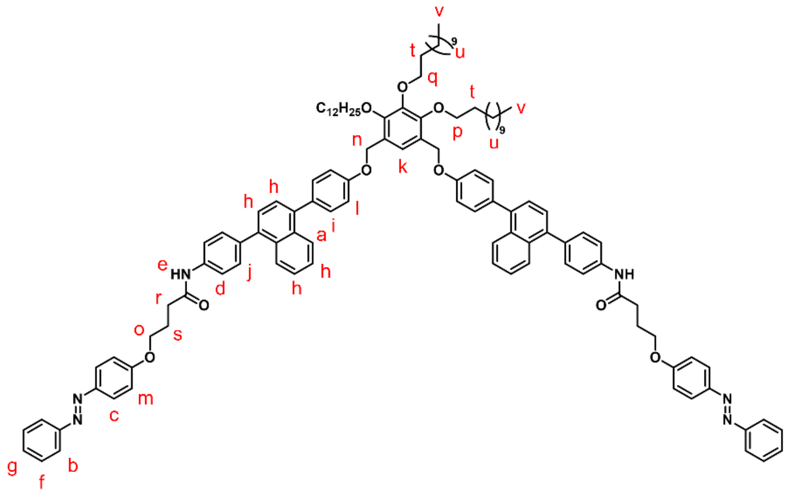

^{1}H NMR (500 MHz, $CDCl_3$, 293K) δ: 8.00–7.91 (m, 8H, NaphH_a, AzoH_b), 7.87 (d, J = 7.7

Hz, 4H, AzoH_c), 7.63 (d, J = 7.8 Hz, 4H, PhH_d), 7.54 (s, 2H, PhNH_eCO), 7.51–7.34 (m, 23H, AzoH_f, AzoH_g, NaphH_h, PhH_i, PhH_j, PhH_k), 7.11 (d, J = 7.9 Hz, 4H, PhH_l), 7.03 (d, J = 8.2 Hz, 4H, AzoH_m), 5.17 (s, 4H, PhCH_n), 4.18 (t, J = 5.3 Hz, 4H, CH_o), 4.13 (t, J = 6.3 Hz, 4H, CH_p), 4.06 (t, J = 6.3 Hz, 2H, CH_q), 2.69 (t, J = 6.7 Hz, 4H, CH_r), 2.34–2.29 (m, 4H, CH_s), 1.82–1.76 (m, 6H, CH_t), 1.52–1.22 (m, 54H, CH_u), 0.89–0.83 (m, 9H, CH_v). ^{13}C NMR (125 MHz, $CDCl_3$, 293K) δ: 170.68, 161.27, 158.32, 152.74, 151.84, 147.11, 145.87, 139.50, 138.75, 136.98, 136.89, 133.22, 132.12, 131.93, 131.19, 130.77, 130.45, 129.07, 129.01, 126.51, 126.47, 126.26, 126.01, 125.84, 125.78, 124.86, 122.61, 119.91, 119.83, 114.73, 114.67, 74.33, 73.76, 67.22, 65.51, 33.99, 31.98, 31.94, 30.50, 30.47, 29.79, 29.76, 29.74, 29.71, 29.64, 29.62, 29.44, 29.40, 26.25, 26.23, 25.05, 22.74, 22.71, 14.17, 14.16. HRMS (APCI): m/z calcd for $C_{120}H_{141}O_9N_6$ 1810.0755 [M+H]$^+$, found 1810.0734.

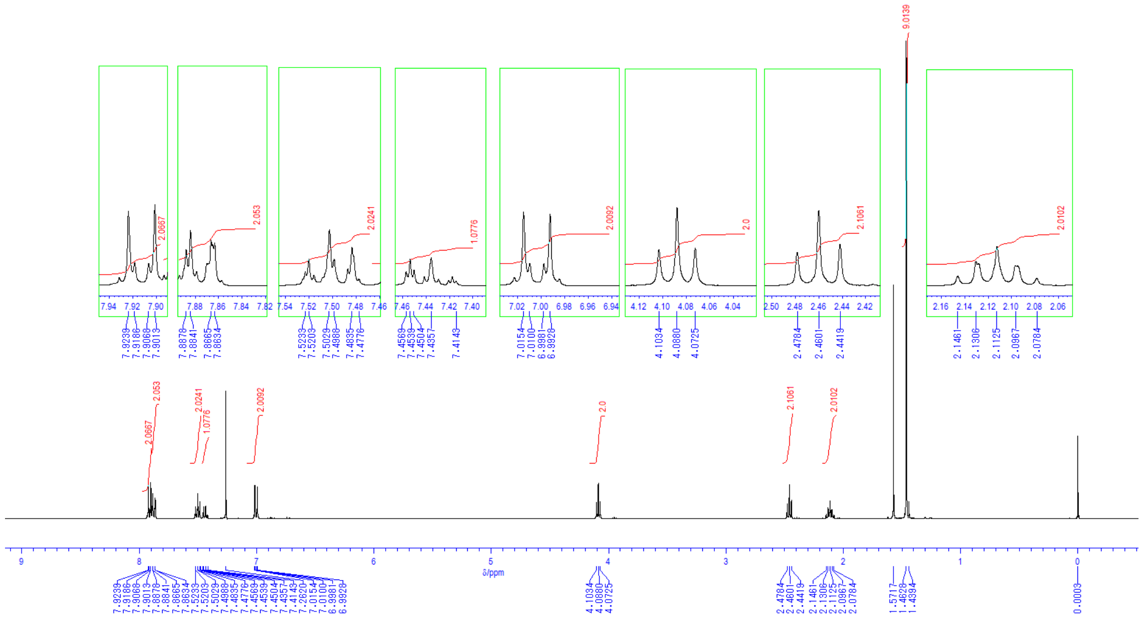

Chart S1. ^{1}H NMR spectrum of **5** in $CDCl_3$ at 293 K.

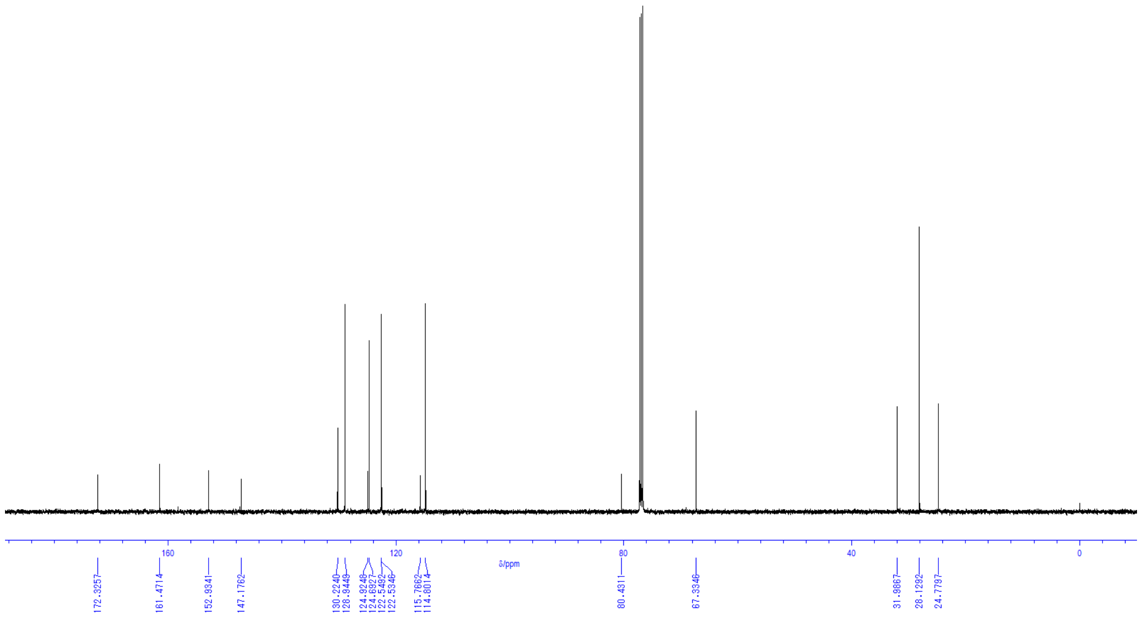

Chart S2. ^{13}C NMR spectrum of **5** in $CDCl_3$ at 323 K.

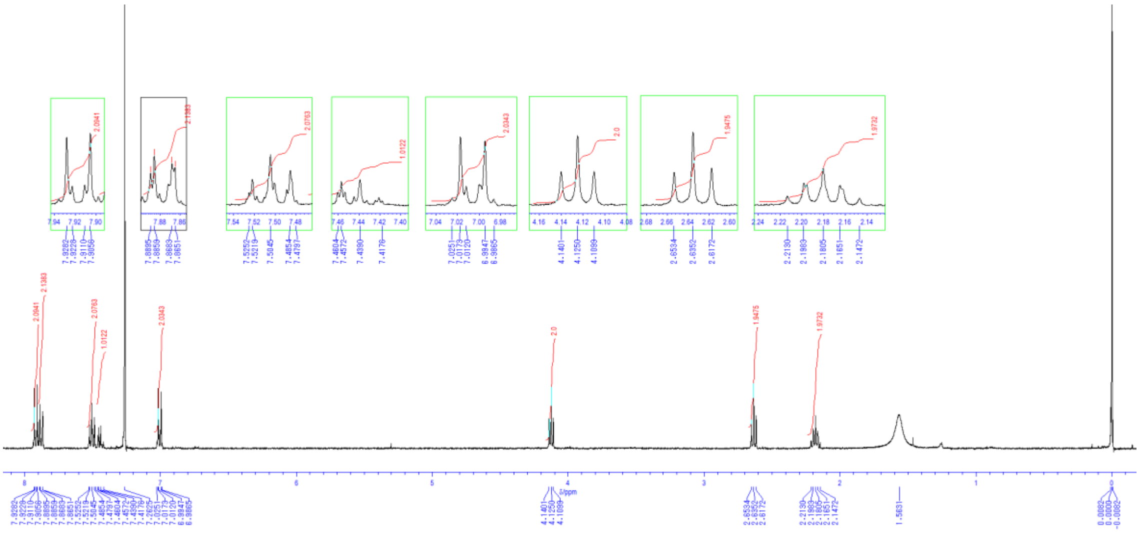

Chart S3. ^{1}H NMR spectrum of **4** in $CDCl_3$ at 293 K.

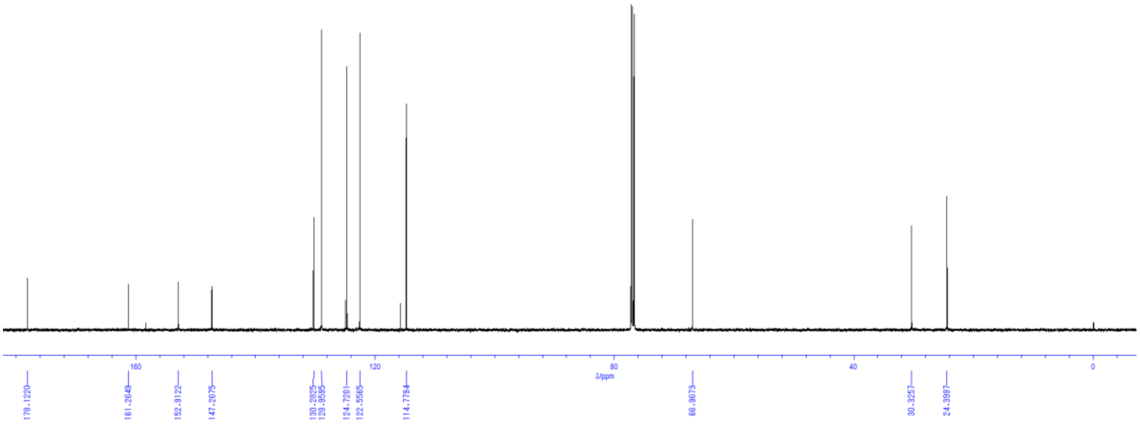

Chart S4. ^{13}C NMR spectrum of **4** in $CDCl_3$ at 323 K.

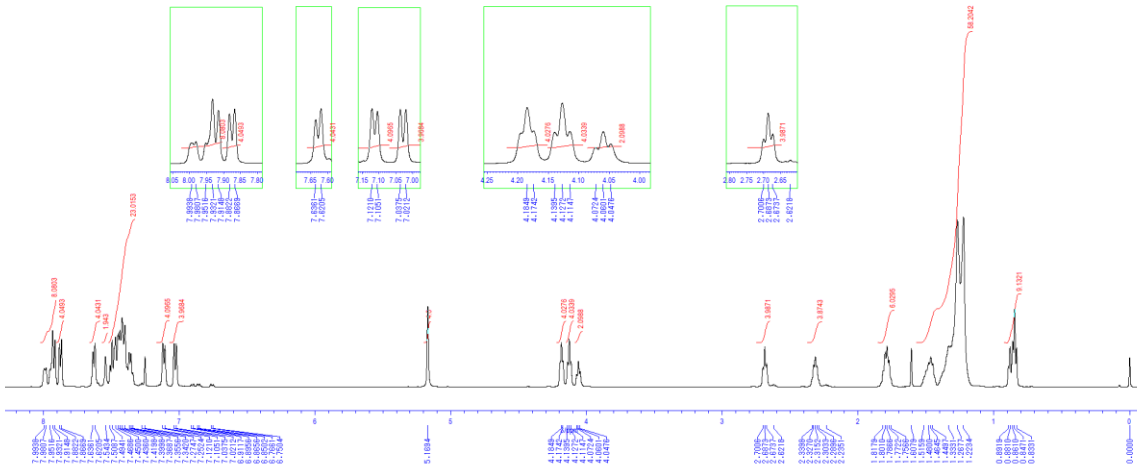

Chart S5. ^{1}H NMR spectrum of **2** in $CDCl_3$ at 293 K.

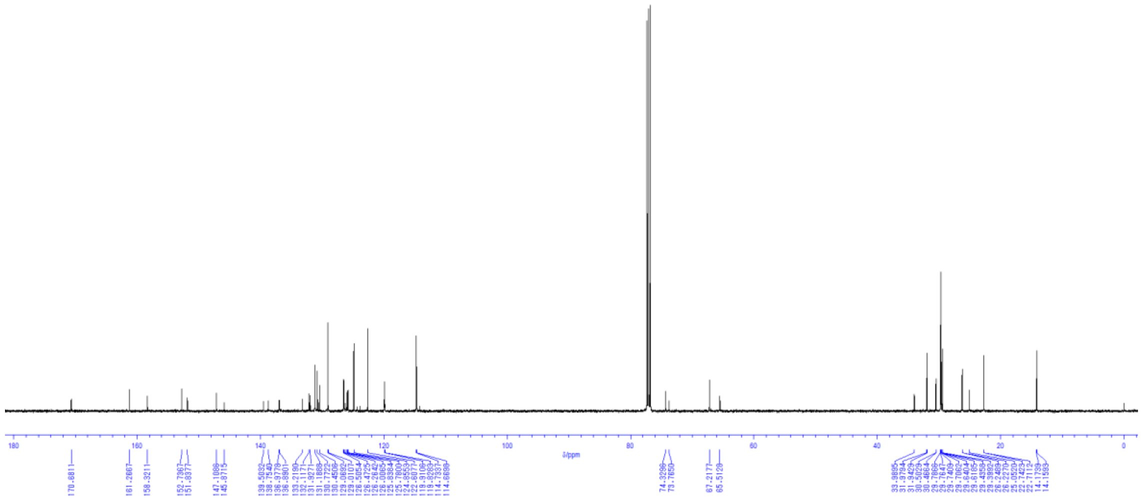

Chart S6. ^{13}C NMR spectrum of **2** in $CDCl_3$ at 293 K.

3. Supporting Figures

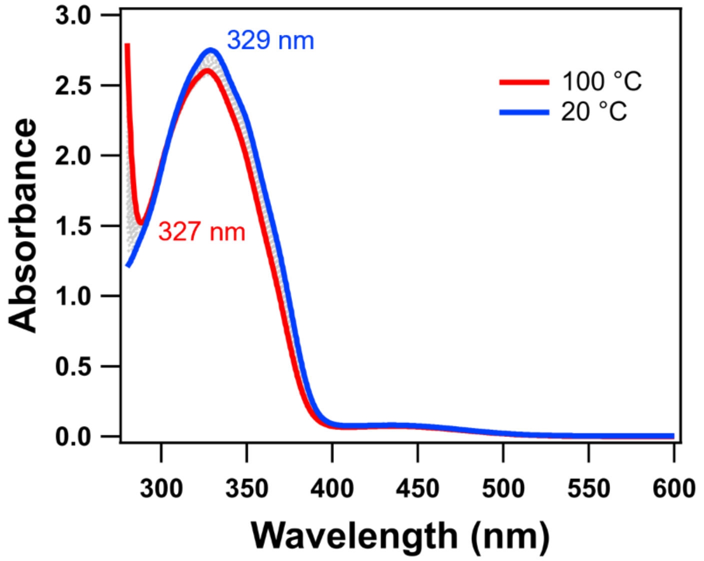

Fig. S1 Changes in the UV–Vis absorbance of **2** in toluene (c = 500 μM) upon cooling from 100 °C (red line) to 20 °C (blue line) at a rate of 1 °C/min. The intermediate spectra were measured at 5 °C intervals.

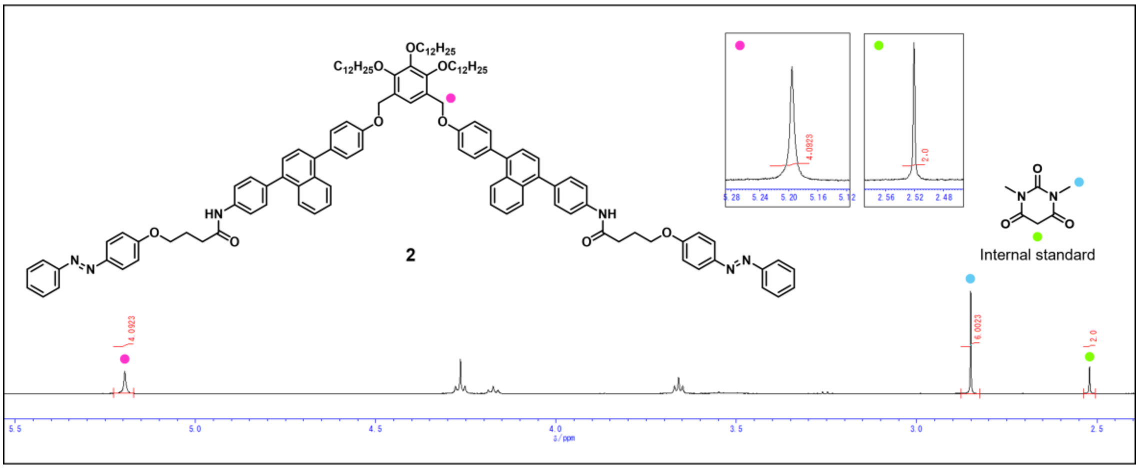

Fig. S2 Partial ^{1}H NMR spectra of a 1:1 mixture (c = 500 μM, each) of **2** and the internal standard, 1,3-dimethyl barbituric acid, in toluene-d_8 at 20 °C. The sample was prepared by cooling the solution of **2** in toluene-d_8 from 100 to 20 °C at a rate of 1 °C/min, and then dissolving the internal standard. *Note*: Because the molar ratio determined from the integration values agrees with the mixing ratio (1:1), it is concluded that **2** does not form NMR-inactive aggregates in toluene (*i.e.*, it exists in a monomerically dispersed state).

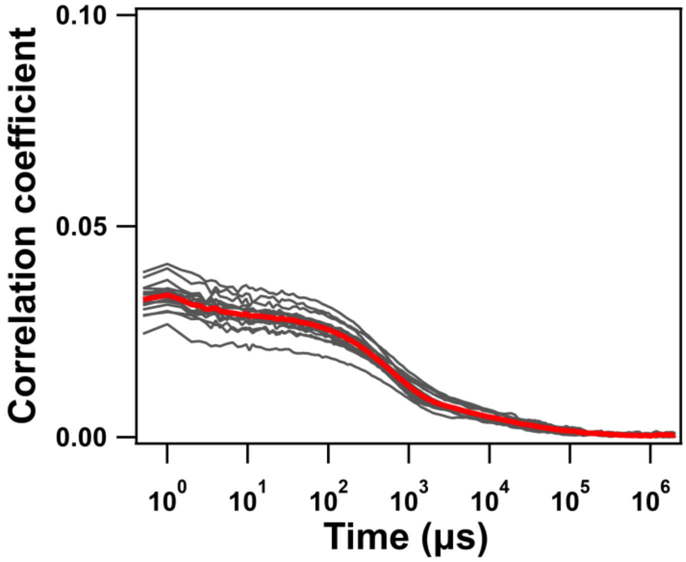

Fig. S3 Raw (gray lines) and averaged (red line) autocorrelation functions obtained by DLS measurements of a toluene solution of **2** at 20 °C. *Note*: The small correlation coefficient (less than 0.05) indicates the absence of any detectable large aggregates in the solution.

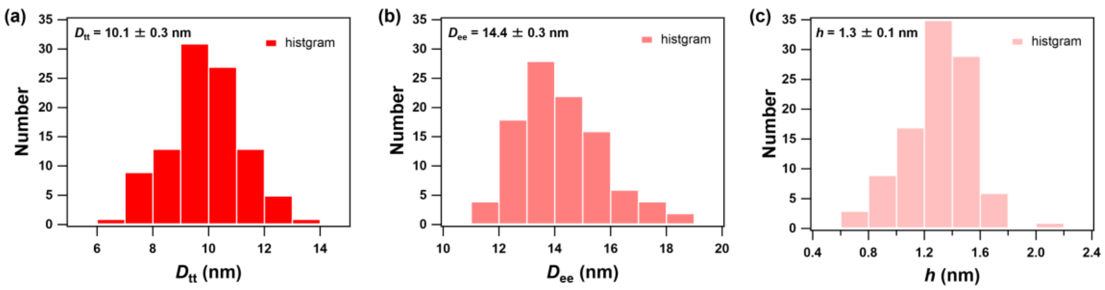

Fig. S4 (a–c) Distributions of the top-to-top diameter, D_{tt} (a), edge-to-edge diameter, D_{ee} (b), and height, *h* (c), of monolayered toroids of **2**.

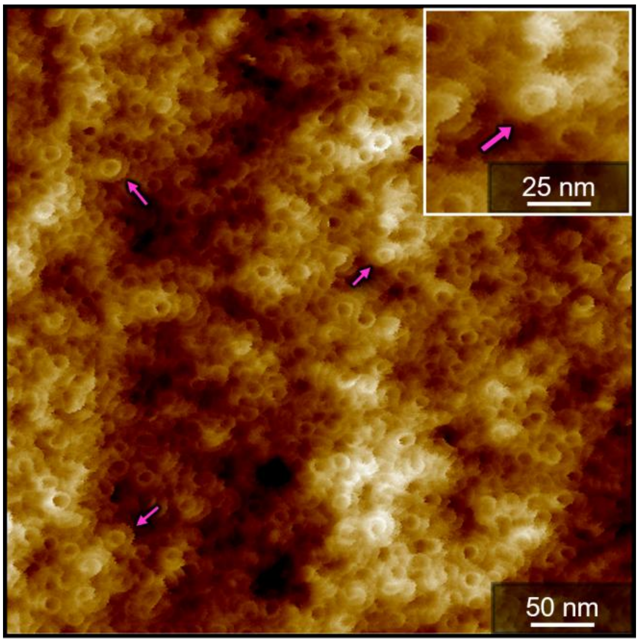

Fig. S5 AFM image of bilayered toroids of **2** formed in toluene (c = 500 μM) upon cooling the solution from 100 to 20 °C at a rate of 1 °C/min. The sample was prepared by drop-casting the solution of **2** in toluene onto an HOPG substrate. Bilayered toroids are highlighted by pink arrows. Inset shows a magnified AFM image of bilayered toroid.

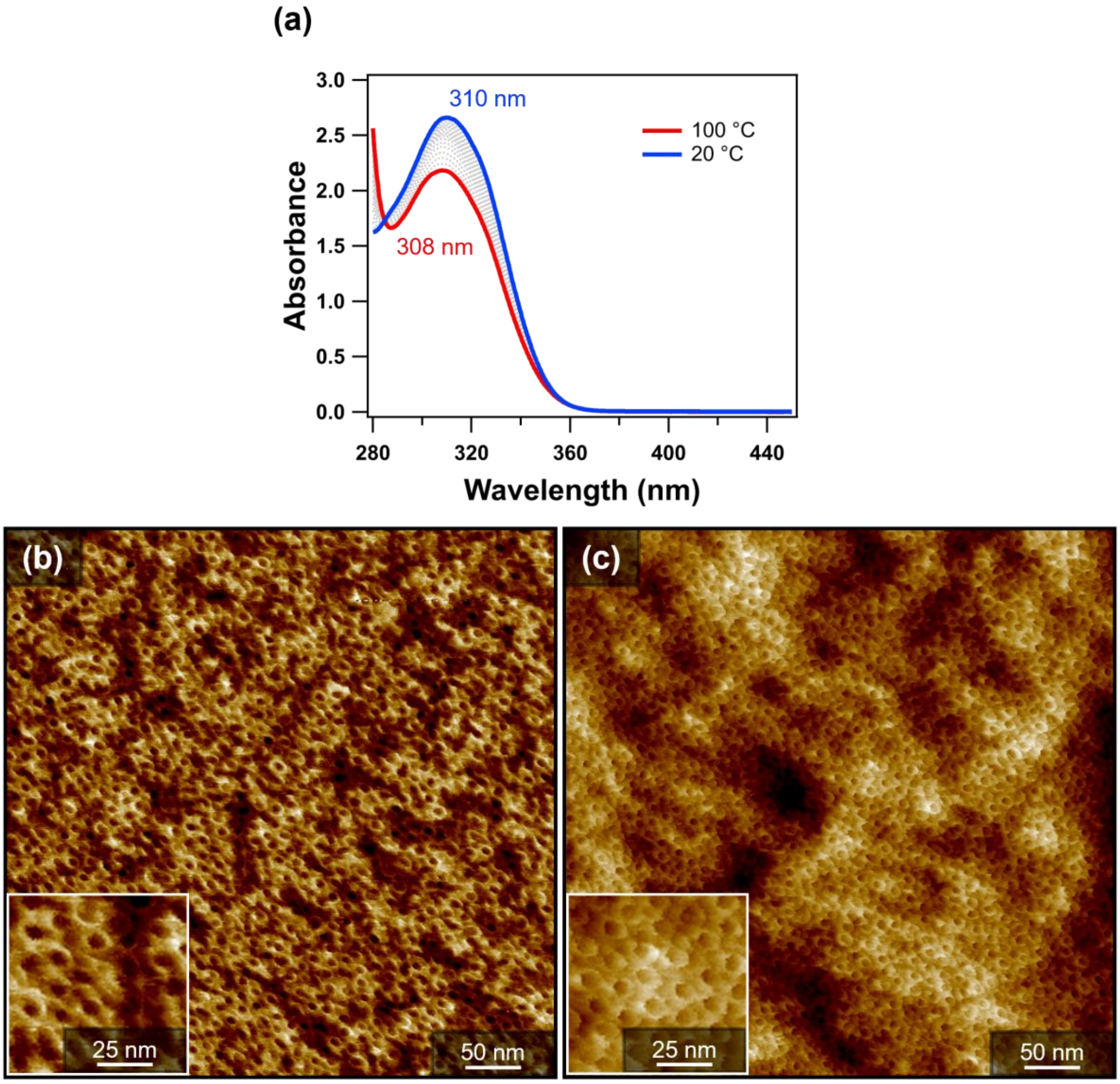

Fig. S6 (a) Changes in the UV–Vis absorbance of **1** in toluene (c = 500 μM) upon cooling from 100 to 20 °C at a rate of 1 °C/min. The intermediate spectra were measured at 5 °C intervals. (b and c) AFM images of monolayered toroids of **1** formed in toluene upon cooling the solution from 100 to 20 °C at a rate of 1 °C/min. The samples were prepared by (b) spin-coating and (c) drop-casting the solution of **1** in toluene onto HOPG substrates. Insets in (b) and (c) show magnified AFM images of monolayered toroids.

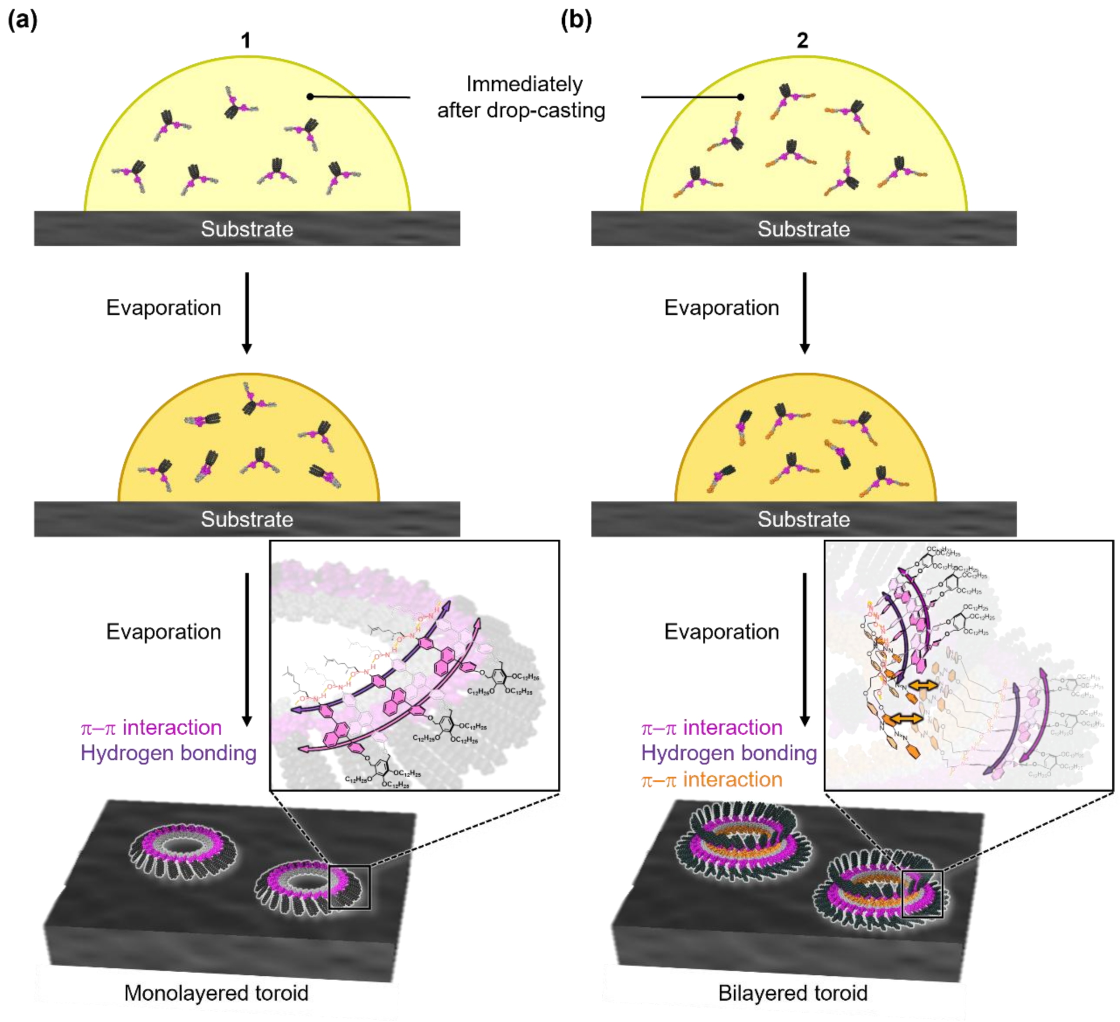

Fig. S7 (a and b) Schematic representations of the distinct self-assembly behaviors of (a) **1** and (b) **2** upon evaporation of the toluene solutions drop-casted onto HOPG substrates.

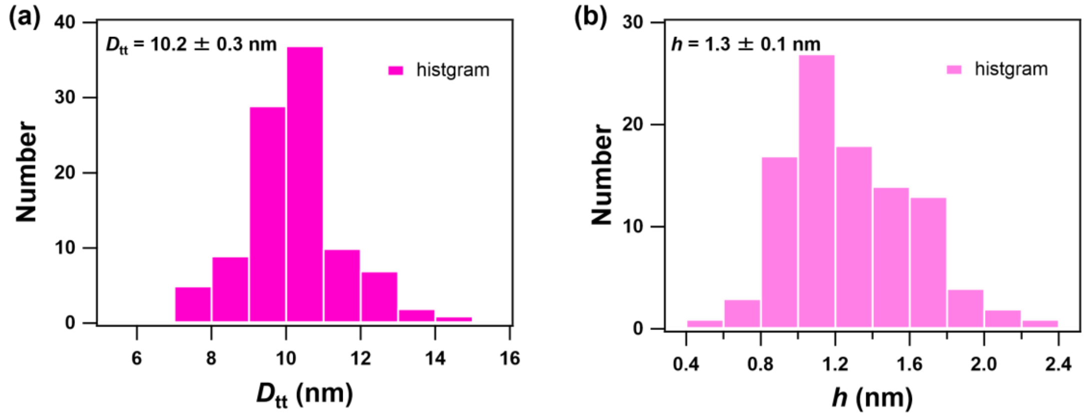

Fig. S8 (a and b) Distributions of the top-to-top diameter, D_{tt} (a), and height, h (b), of bilayered toroids of **2**.

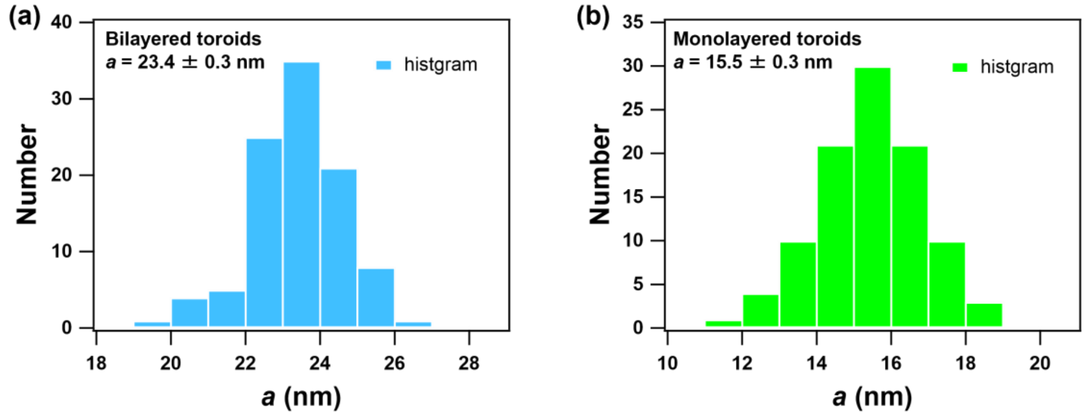

Fig. S9 (a and b) Distributions of the lattice parameter, a, for assemblies of bilayered toroids (a) and monolayered toroids (b) of **2**.

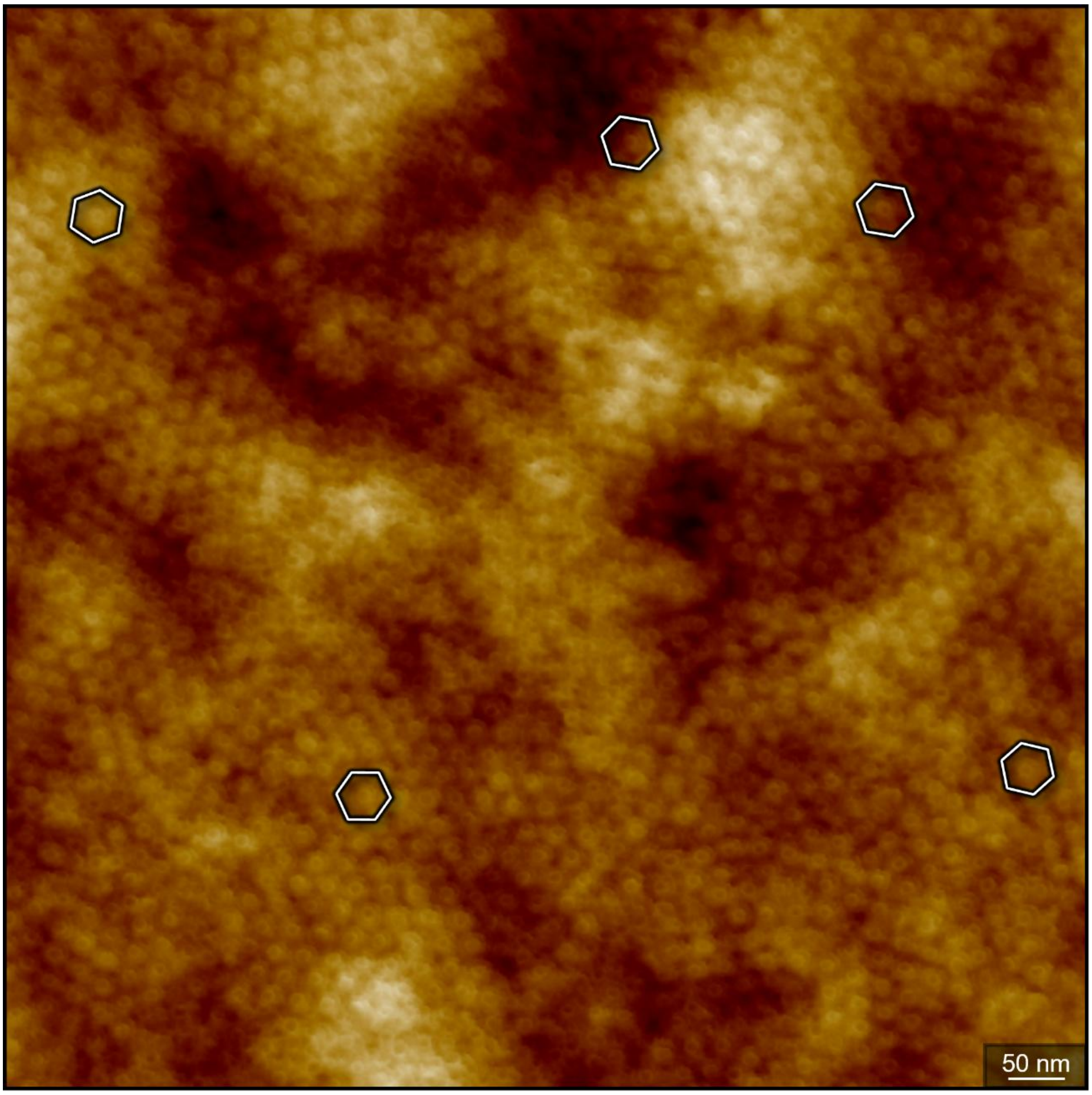

Fig. S10 Large-area AFM image of the surface of a micro-sized sheet dispersed in toluene. The bilayered toroids of **2** exhibit hexagonal packing that extends over a wide area while maintaining structural ordering across surface height variations.

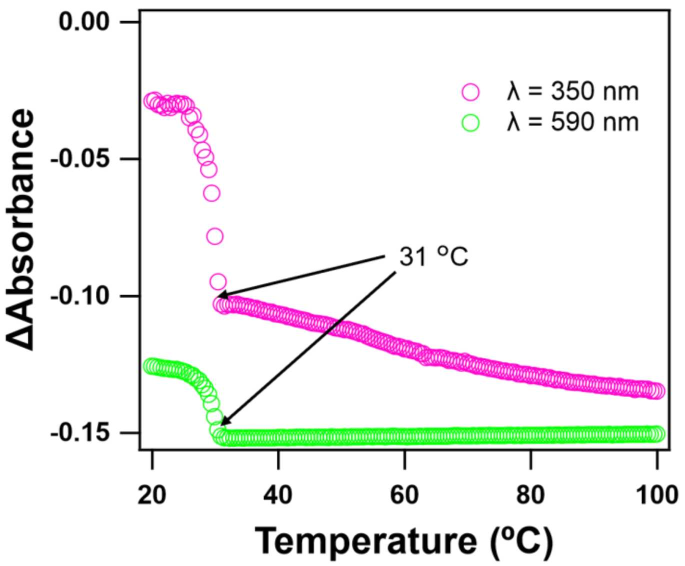

Fig. S11 Cooling curves of **2** in MCH (c = 10 μM) obtained by temperature-dependent absorption measurements at 350 nm (pink) and 590 nm (light green). The cooling rate was 1 °C/min.

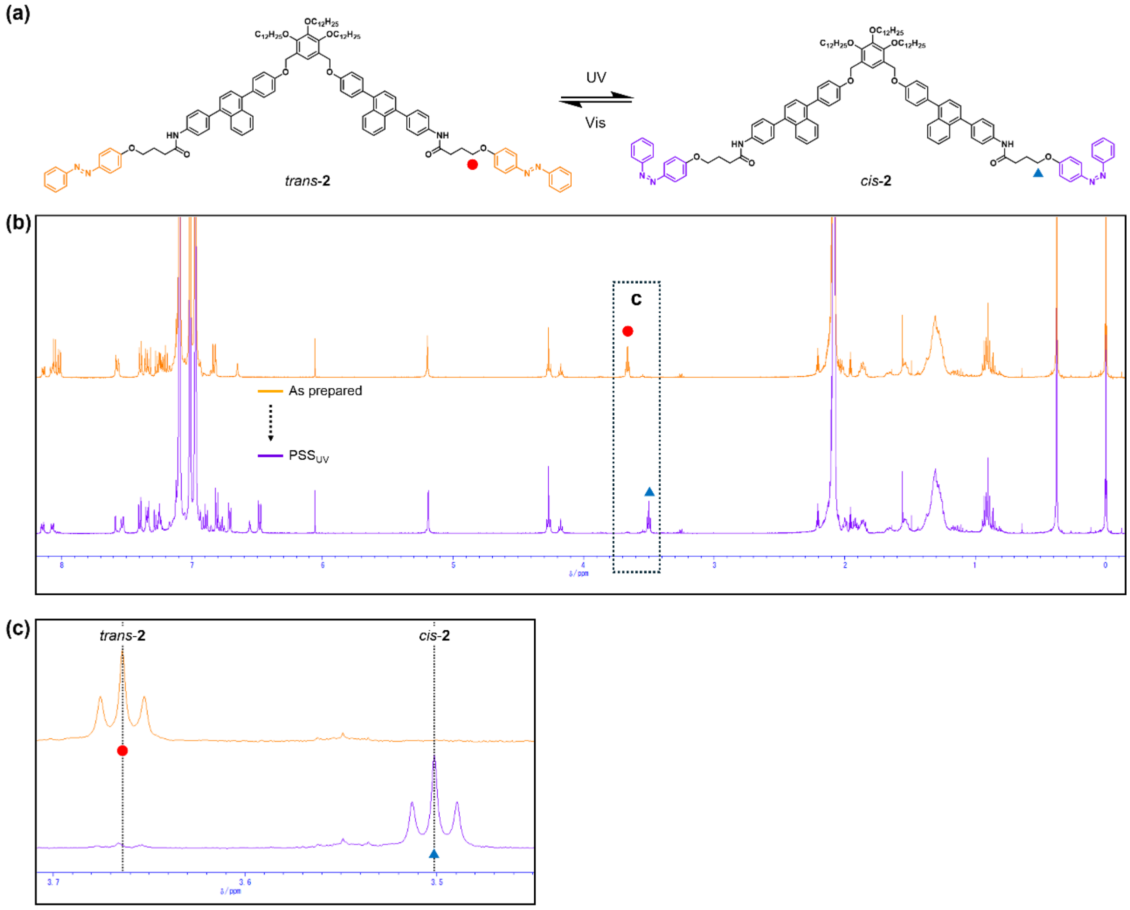

Fig. S12 (a) Chemical structures of *trans*-**2** and *cis*-**2**. (b) ^{1}H NMR spectra of **2** in toluene-d_8 (c = 500 μM) at 20 °C before (orange) and after irradiation with UV light (λ = 365 nm) for 5 min (PSS$_{UV}$, purple). (c) Magnified view of the ^{1}H NMR region enclosed by the dotted box in (b). *Note*: The disappearance of the peak assigned to *trans*-**2** (shown by the solid red circle) after UV irradiation indicates that the photoisomerization proceeded almost quantitatively.

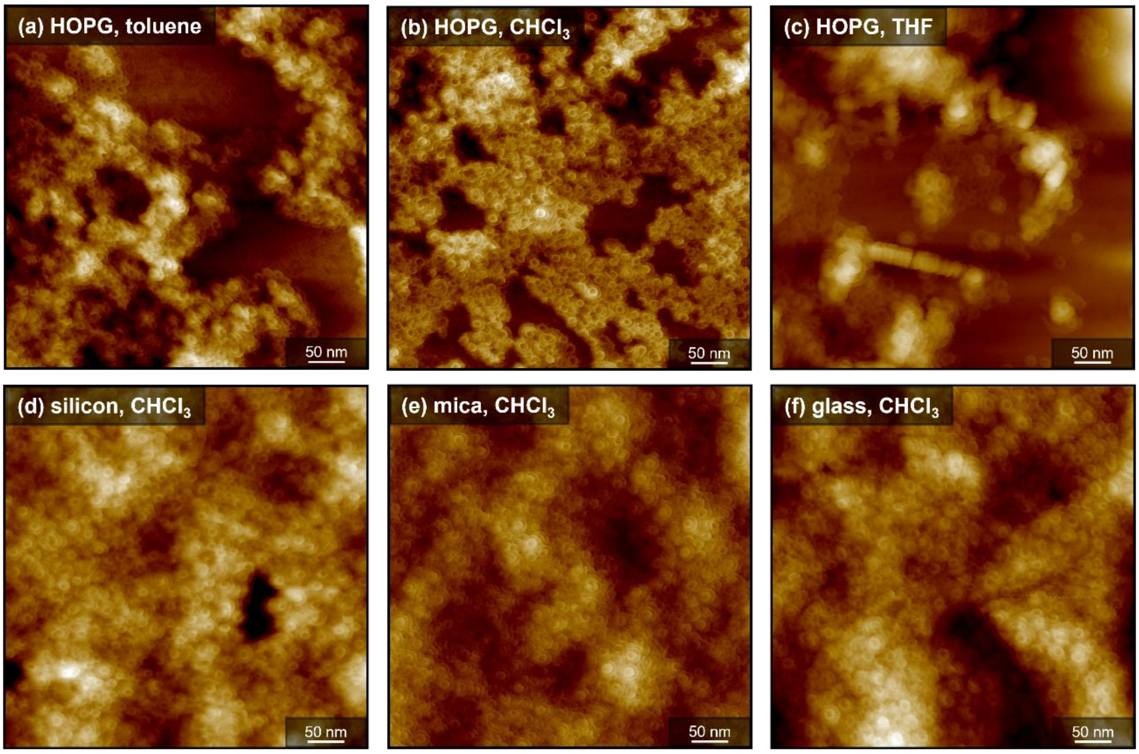

Fig. S13 (a–f) AFM images of bilayered toroids of **2** (c = 100 μM) formed in different solvents: toluene (a), $CHCl_3$ (b, d–f), and THF (c). The samples were prepared by drop-casting the solutions onto HOPG (a–c), silicon (d), mica (e), or glass (f).

4. Supporting References

S1. T. Aizawa, H. Arima, S. Mihara, T. Ueno, S. Yoshii, T. Saito, H. Itabashi, S. Datta, H. Hanayama, A. Sakamoto, R. Shimada, S. E. Rogers, M. J. Hollamby, T. Kajitani, Y. Ishii, G. Watanabe, K. Harano, T. Matsumoto, N. Pathoor, M. Vacha, H. Sotome and S. Yagai, *J. Am. Chem. Soc.*, 2026, jacs.6c00854.
